# The Sequelæ of Suppurative Otitis Media
*This paper is—with a few additions due to later researches—a thesis read for the degree of M.B. (Cantab) in Easter Term of 1889. It is an attempt to summarise our present knowledge of the various complications which may result from suppurative otitis media.


**Published:** 1890-03

**Authors:** F. H. Edgeworth

**Affiliations:** Physician to the Bristol Hospital for Sick Children and Women; Scholar of Cains Coll., Cambridge; Late Medical Tutor, Bristol Medical School


### THE SEQUELÆ OF SUPPURATIVE OTITIS MEDIA*

BY

F. H. Edgeworth, B.A., M.B. (Cantab), B.Sc. (Lond.),

*Physician to the Bristol Hospital for Sick Children and Women; Scholar of Cains Coll., Cambridge; Late Medical Tutor, Bristol Medical School.*

---

The chief interest of suppuration in the middle ear arises from its liability of spreading to neighbouring important structures. The determining factors of this are the intimate relations of the lining mucous membrane with the circumjacent bone; for the deeper layer of this forms the endosteum, and sends processes into the bone, so that an inflammatory change is most easily propagated to the bone. The causes of this propagation lie chiefly in the want of free removal of inflammatory products of the mucous membrane: though the membrana tympani be perforated, the tympanic cavity is at the bottom of a long narrow channel, so that stagnation readily occurs; and once inflammation has spread to the bone, the carious and necrotic products are with difficulty discharged, and the inflammatory change progresses further. Such a process would be of less importance in any other bone, but the petrous portion of the temporal bone occupies a unique position in that it has relations with important

* This paper is—with a few additions due to later researches—a thesis read for the degree of M.B. (Cantab) in Easter Term of 1889. It is an attempt to summarise our present knowledge of the various complications which may result from suppurative otitis media.

structures. Thus a very thin roof separates the tympanic from the cranial cavity opposite the under surface of the temporo-sphenoidal lobe of the brain. A thin plate of bone anteriorly separates the middle ear from the carotid canal. On the inner side, two membranes only separate the tympanic cavity from the internal ear, whence there is easy communication with the cranial cavity. Posteriorly the tympanic cavity opens, at all ages of life, into the mastoid antrum; while in adults the large mastoid process, often with pneumatic spaces communicating with the antrum, readily becomes the seat of consecutive morbid changes. The mastoid antrum is close to the lateral sinus, only separated by a thin lamina of bone. Owing to these relations it will be evident that inflammatory change has but to spread a little way through the walls of the tympanic cavity to light up most serious mischief.

It is the object of this paper to treat of the diagnosis and principles of treatment of the various sequelæ which arise from these extensions of middle-ear suppuration.

Some of these sequelæ have long been known, and are readily diagnosed and treated; others, though long known as sequelæ, have only within the last two or three years been recognised during life and successfully treated. This is more especially true of brain abscess.

These sequelæ may be enumerated, as follows:

1. Pent-up pus in the middle ear, with occasionally supramastoid abscess.

2. Caries of either the petrous or mastoid portions of the temporal bone. Polypi.

3. Abscess between the dura mater and temporal bone.

4. Bleeding from the carotid artery.

5. Thrombosis of the carotid artery.

6. General septic meningitis.

7. Thrombosis of cerebral sinuses, with septicæmia or pyæmia.

8. Cerebral abscess.

9. Cerebellar abscess.

These sequelæ are, with the exception of 4 and 5, put down much in the order in which they are likely to occur in cases of chronic suppurative otitis media. In acute suppurative otitis media brain abscess rarely forms, caries does not occur so commonly, whilst pent-up pus in the middle ear, meningitis, and thrombosis of sinuses are very frequent.

1. *Pent-up pus in the middle ear, with occasionally supramastoid abscess.*—In cases of acute suppuration in the middle ear, the membrana tympani may not readily give way. In such a case, meningitis or sinus thrombosis, or both, readily occurs from extension of inflammation inwards; but, apart from this, severe symptoms may arise from retention of the products of an acute suppuration in the middle ear, such as hardly to be distinguished from those of septic meningitis, and yet proved not to be due to this by their subsidence on perforation of the membrana tympani. Such symptoms may be: great pain in the ear, tinnitus, vertigo, delirium, associated with fever and often severe rigors. And in one or two cases it is said that optic neuritis has occurred. The explanation of this last symptom is very difficult; and, indeed, the cerebral symptoms are such that, although it is not possible to suppose that suppurative meningitis occurs in these cases, yet they seem to indicate some special cerebral irritation, possibly of the nature of a meningeal sapræmia. The treatment of such cases of acute suppurative otitis media with retention of pus is easy: on inspection, the membrana tympani is seen bulging and opaque; on incising this, preferably in the postero-inferior sector, pus will escape. The middle ear should then be well syringed out. The symptoms due to the pent-up pus will soon abate; and if meningitis or sinus-thrombosis has not occurred, the patient will quickly recover.

In such cases of pent-up pus in acute suppurative otitis media, inflammation may spread, probably through lymphatic channels, outwards along the walls of the external auditory meatus to the tissues over the mastoid process, and an abscess will form. There will first be a painful swelling of the tissues behind the ear, which may extend over the upper part of the side of the neck. Then fluctuation will be detected over the mastoid process. Such an abscess is accompanied by fever, which only subsides on the bursting or evacuation of the abscess. It may be somewhat difficult, before operation, to diagnose such a supramastoid abscess from an acute suppurative periostitis of the mastoid process: the former, however, only occurs in those cases of acute otitis media where the membrana tympani does not readily give way; whilst the latter generally, though not always, occurs in the course of a chronic otitis media which has spread to the mastoid cells. On operation, however, the distinction is easy, if the finger be introduced: in the supramastoid abscess, the mastoid process is felt in a perfectly normal condition forming the floor of the abscess. Such an abscess will quickly close on incision and drainage.

2. *Caries and necrosis of the temporal bone.*—The causes of this have been already stated, together with the effects of its spreading towards the interior of the skull. The inflammatory change undergone by the bone is a chronic septic suppuration—caries and necrosis take place. This carionecrosis will in many cases be confined to the inner wall, affecting the promontory and the Fallopian aqueduct: in such cases there will rarely be any blocking of the discharge, the nature of which is characteristic; it is a cream-coloured offensive fluid, containing larger or smaller particles of bone in suspension. Often this discharge is the only sign that the bone is affected—the pain is slight, or more generally absent, and there is no febrile disturbance. The inflammation may spread to the Fallopian aqueduct, causing paresis or paralysis of the facial nerve, with loss of taste in the anterior two-thirds of the tongue.

The treatment of such uncomplicated cario-necrosis of the tympanic walls is simple and generally successful. Its principles are: the complete removal of secretions, and the application of antiseptics to the carious surface. It is sometimes needful to trephine the mastoid, so as to get free drainage of the middle ear: if this be done, the most obstinate cases will generally quickly heal.

The discharge through the auditory meatus is in such cases often partially blocked by *polypi*, which are chiefly of importance on this account. They can easily be removed by a pair of forceps or sharp spoon.

The chance of stagnation of discharge becomes much greater if the inflammation attack the antrum and the mastoid process. If the discharge is unimpeded, the inflammation gives rise to no special symptoms; but if retention takes place, reactive phenomena arise. If the inflammation does not spread outwards to the surface, there will be no mastoid swelling; but tenderness on pressure over the mastoid will exist, together with pain in the mastoid region, and fever with possibly some signs of cerebral irritation. If the inflammation progress outwards, there will shortly arise a swelling behind the ear, hot and tender to the touch—at first hard, afterwards fluctuating: an incision being made, pus and bony *débris* will be found as an abscess beneath the periosteum; by slight scraping, communication is easily made with the middle ear. Under such drainage, healing soon takes place.

The term "trephining the mastoid" might be reserved for those cases in which, to reach the pent-up pus in the mastoid, no external guide in the shape of a subperiosteal swelling exists. The great danger in such an operation is injury to the lateral sinus: in order to avoid this, the mastoid should be trephined immediately behind the posterior border of the opening of the auditory meatus, and not higher up than its superior border; and gouging should take place forwards and inwards, *i.e.* parallel with the meatus. In such a direction the bone may be safely pierced to the depth of $1\frac{1}{2}$ c.m., within which distance the pus is almost invariably found, and communication with the middle ear opened up by way of the mastoid antrum. The result of such trephining operations has been most uniformly successful, the suppuration ceasing on the thorough drainage. In rare cases pus pent up in the mastoid does not extend externally, but downwards, so as to burst on the infero-internal surface of the mastoid, *i.e.* beneath the deep cervical fascia. This leads to a painful infiltration of the side of the neck, with formation of abscess.

3. *Abscess between the temporal bone and dura mater* ("Subdural" of Barker).—Cario-necrosis of the mastoid cells may in some cases extend inwards, leading to the formation of pus between the bone and dura mater. This is more particularly apt to occur in chronic badly-drained cases.

The pus in such cases is usually found either over the roof of the tympanum, close to the squamo-petrous suture, or on the posterior surface of the petrous bone, *i.e.* in the groove for the lateral sinus; or it may extend from one to the other of these places.

The symptoms point to the local suppuration: there will be fever, and the patient will complain of pain over the temporal bone; and there will be found tenderness and œdema over the mastoid or squamous portions of the temporal bone.

This will lead to trephining the mastoid; but it will be found that the symptoms persist after this, for the pus is not drained away. If this occurs, the two following operations must be done: first, gouging the squamous portion of the temporal bone ½ in. above and behind the auditory meatus; and if pus is not found there, gouging the mastoid ½ in. directly behind the auditory meatus. If pus underlie the bone, this will very probably be found spongy, and pus will ooze out before the abscess is reached.

The evacuation of the pus will lead to the prompt abatement of the symptoms.

4. *Hæmorrhage from the carotid artery.*—A few instances have been recorded of caries of the temporal bone progressing forwards, opening up the carotid canal and causing bleeding from the internal carotid artery. The symptoms will be sufficiently obvious: bright arterial blood will issue from the meatus synchronously with the pulse.

Ligature of the internal carotid is of very little use, by reason of the intracranial and extracranial anastomoses of the artery. Plugging the meatus may arrest the bleeding for a time. Such cases have been uniformly fatal.

5. *Thrombosis of the internal carotid artery.*—A unique case has been recorded by Gairdner, in which caries of the petrous bone from middle ear disease caused thrombosis of the internal carotid artery, and so minute embolic lesions of that side of the brain. The symptoms were: sudden rise of temperature to 103°, hemiplegia, and progressive coma—going on to death within twenty-four hours.

6. *General meningitis.*—The ear disease which gives rise to meningitis is much more often acute than chronic, in this offering a marked contrast with cerebral abscess. The inflammation is a lepto-meningitis—a septic purulent inflammation of the pia-arachnoid, which is set up by direct extension from the septic focus in the middle ear, either through the tympanic roof, or by the internal ear, or through the mastoid cells. The inflammation may be limited to the convexity of the brain, or, more rarely, to the base; most commonly it becomes general.

Of the symptoms, some are independent of the exact locality of the meningitis: of these the more important are, headache, delirium, vomiting, general convulsions, hyperæsthesia of skin and special senses, optic neuritis. *Headache* may be frontal or general; it may undergo exacerbations, causing a characteristic shriek or cry; it is characteristic of the headache that it does not cease as delirium comes on, but persists until the patient becomes comatose. The *delirium* may be noisy or quiet; it is usually well marked. *Vomiting* is generally an early symptom; it has all the characteristics of "cerebral" vomiting. General *epileptiform convulsions* occur whether the meningitis be localised or general. *Hyperæsthesia of skin*, and more particularly of ear and eye, occur, so that, *e.g.*, the slightest sound or light causes pain.

It is questionable whether optic neuritis is a general cerebral symptom; it occurs but rarely when the meningitis is confined to the convexity of the brain, whereas it is very common if the meningitis be basal. If the meningitis be basal, there occur characteristic symptoms: contraction or inequality of the pupils, with ptosis and defect of ocular movements, due to involvement of the oculo-motor nerves by the inflammation; if the meningitis spreads backwards down the cord, there arise symptoms of cervical meningitis.

A meningitis of the convexity of the brain will affect the motor areas, causing muscular rigidity, hemiplegia or paraplegia, with convulsions unilateral or bilateral.

*Pyrexia* is always present, and may reach a high degree, *e.g.* 104° or 105°, within a few hours of the onset of the meningitis; and it remains high, often running up still higher before death. The *pulse* is very variable, most often frequent, but bearing no constant proportion to the temperature. The course of such a general purulent septic meningitis is very acute; it invariably destroys life in a few hours or days. The symptoms of cerebral irritation—headache, delirium, convulsions—pass into a fatal coma.

The differential diagnosis of meningitis has to be made from (1) acute pent-up suppuration in the middle ear—this has been already treated of; from (2) acute temporo-sphenoidal and cerebellar abscess—this will be referred to later on; from (3) sinus-thrombosis—this often co-exists with meningitis, but when absent may be excluded by the absence of extracranial extension of clot and of secondary abscesses.

7. *Thrombosis of cerebral sinuses, with septicæmia or pyæmia.*—This may be a sequel of either acute or chronic middle ear suppuration. The veins of the middle ear pass in two directions—into the temporo-maxillary vein, and into the superior petrosal sinus; whilst from the mastoid cells veins pass into the lateral sinus. Hence, from the middle ear or mastoid cells, septic thrombosis of the veins readily spreads into these sinuses. Such an event, though not dependent on, is yet often associated with or preceded by caries of the temporal bone; for the conditions which give rise to each are the same, *i.e.* retention of inflammatory products.

Thrombosis of the superior petrosal sinus readily spreads upwards to its tributary cerebral veins, forwards to the cavernous sinus and ophthalmic vein, and backwards to the lateral sinus. Thrombosis of the lateral sinus spreads to the mastoid emissary vein and to the internal jugular vein. The clot is septic; hence, whether or not there be an accompanying meningitis, this latter condition often arises secondarily to the thrombosis, by spreading of inflammation through the sinus walls.

From the same reason, general septic inflammation of the blood usually takes place, causing septicæmia or pyæmia. In about one-half of the cases there are secondary purulent deposits—most often in the lungs.

The effects of the sinus-thrombosis on the cranial circulation and brain will be small, by reason of the free anastomoses of the cerebral veins. Thrombosis of the cavernous sinus and ophthalmic vein will cause proptosis, œdema of eyelids and conjunctiva, œdema of face with dilitation of facial veins (due to the diversion of the orbito-facial circulation), together with supraorbital neuralgia and defect of ocular movements from interference with the nerves round the cavernous sinus. If the clot extend to the opposite cavernous sinus, similar effects will appear on the other side of the face. The effects of extension of the thrombosis to the mastoid emissary vein will be tenderness and œdema over the mastoid foramen. A clot in the internal jugular vein will cause tenderness and œdema extending down its course.

Septicæmia or pyæmia will be evidenced by rigors, with fever, remittent or intermittent, profuse sweating, typhoid aspect, together with signs of abscesses, if metastatic deposits take place.

Meningitis will be evidenced by its ordinary signs: high temperature, headache, vomiting, various paralyses, with general or localised convulsions, ending in coma and death.

In any case, then, of sinus-thrombosis the symptoms may vary to a great degree: they may be chiefly those of extracranial extension of clot with those of pyæmia or septicæmia; or there may be those of septicæmia or pyæmia, but no signs of extracranial extension of clot; may be chiefly those of meningitis with extracranial extension of clot, and with or without those of septicæmia or pyæmia. The difficulty of diagnosis will correspondingly vary.

In any case, however, in which there are no signs of extracranial extension of clot or of secondary abscesses, the mastoid should be trephined and thorough drainage of the middle ear thus secured; for mastoid retention may give rise to almost indistinguishable symptoms. If after this the symptoms persist and point unmistakably to sinus thrombosis, the prognosis is hopeless; for the focus is a septic clot which cannot be got at.

*(To be continued.)*

